# 805. Did Mask Mandates have an Affect on Pulmonary Mycoses? Trends of Coccidioidomycosis Incidence during COVID-19 Pandemic Era in California and Texas

**DOI:** 10.1093/ofid/ofad500.850

**Published:** 2023-11-27

**Authors:** Nirusha Abeydeera, Linda Pham, Andrew Hwang, Napatkamon Ayutyanont

**Affiliations:** Riverside Community Hospital/ HCA Healthcare, Riverside, California; Riverside Community Hospital/ HCA Healthcare, Riverside, California; UCR School of Medicine, Riverside, California; HCA Healthcare, Riverside, California

## Abstract

**Background:**

Coccidioidomycosis is a fungal infection endemic to areas in the Southwest United States. This fungus grows in dust and soil; and transmission to humans is through inhalation of spores. To prevent transmission, the CDC recommends avoidance of dusty excavation or construction sites. Furthermore, OSHA recommends use of N95 masks for such work environments. During the winter of 2020, there was rapid worldwide transmission of the novel COVID-19 virus. In response to the pandemic, government agencies implemented mask mandates to reduce transmission of infectious droplets; however, these policies varied by region. This study aims to assess incidence of Coccidioidomycosis infections while mask wearing is prevalent. We hypothesize a reduction of overall infections, during and post pandemic era.

**Methods:**

This is a retrospective cross-sectional study, comparing adults (age 18 and over) hospitalized with Coccidioidomycosis infections from January 2019 to September 2021 in HCA facilities in California and Texas. Infection prevalence was recorded for pre-pandemic, during, and post- pandemic eras, correlating to when each state enforced and ceased mask mandates. Infection rates were compared using Chi-square analysis. A total of 611 patients were included in the study; all with ICD-10 diagnosis of Coccidioidomycosis. Patients with prior coccidioidomycosis or chronic infection were excluded.

Pandemic date ranges for each state

**Table 1:**
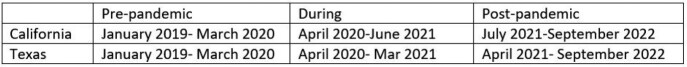
Date ranges for pre-pandemic, during and post-pandemic eras in California and Texas.

**Results:**

There is significant difference in frequency of Coccidioidomycosis admissions, with less admissions post-pandemic (36.9%, p< 0.05) than pre-pandemic (63%) and during (61.6%) in California; and more admissions post-pandemic (63.1%, p< 0.05) than pre-pandemic (37%) and during (38.4%) in Texas.

Coccidioidomycosis Incidence

**Chart 1:**
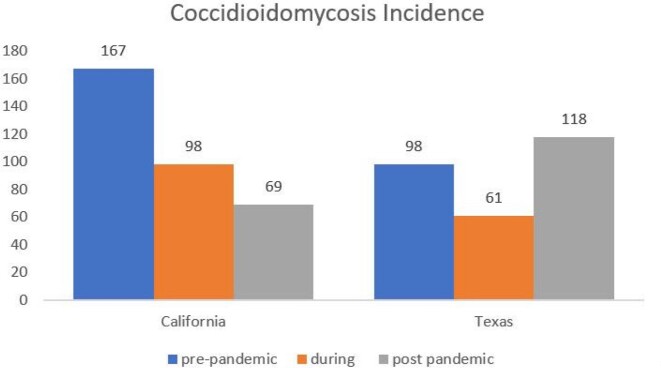
Incidence rates in California and Texas. Data included is number of admissions with Coccidioidomycosis infections as diagnosis. Both post-pandemic admissions are significantly different in comparison to pre-pandemic and during pandemic eras

Incidence rates of Coccidioidomycosis infections

**Table 2:**
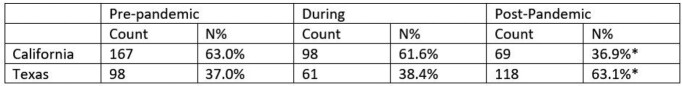
Incidence rates for Coccidioidomycosis infections for pre-pandemic, during, and post-pandemic eras in California and Texas. * suggest significant difference between N% of the same row, p<0.05.

**Conclusion:**

The data reflects reductions of Coccidioidomycosis infections during times of masking in California and Texas. Although OSHA standards are in place for N95 mask wear for reduction of infection, there are no mask wearing recommendations for the general public. With the results noted, masking should be considered effective prevention for populations at risk for infection of Coccidioidomycosis. One limitation of this study was patients were all selected from within the HCA hospital system. A larger study would be beneficial to generalize these results nationwide.

**Disclosures:**

**All Authors**: No reported disclosures

